# Artificial Intelligence in Early Breast Cancer Detection: A Systematic Review of Innovations in Preventive Women’s Healthcare

**DOI:** 10.3390/healthcare14121674

**Published:** 2026-06-12

**Authors:** Anastasia Bothou, Angeliki Bolou, Konstantinos Dinas, Giannoula Kyrkou, Deniece Hardy, Panagiota Pappou, Pinelopi Varela, Georgia Margioula-Siarkou, Myrsini Balafouta, Athina Diamanti

**Affiliations:** 1 Department of Midwifery, University of West Attica, 122 43 Athens, Greece; abolou@uniwa.gr (A.B.); ikirkou@uniwa.gr (G.K.); pappougiota1@uniwa.gr (P.P.); adiamanti@uniwa.gr (A.D.); 2 Gynecologic Oncology Unit, 2nd Department of Obstetrics and Gynecology, Aristotle University of Thessaloniki, 546 42 Thessaloniki, Greece; konstantinosdinas@hotmail.com (K.D.); gmargioulasiarkou@gmail.com (G.M.-S.); 3 Department of Midwifery, University of Greenwich, London SE10 9LS, UK; d.a.hardy@greenwich.ac.uk; 4 Department of Midwifery, International Hellenic University, 570 01 Thessaloniki, Greece; pvarela@uniwa.gr; 5 Department of Biomedical Sciences, University of West Attica, 122 43 Athens, Greece; mbalafouta@uniwa.gr

**Keywords:** artificial intelligence, breast cancer screening, women’s health, preventive healthcare, early detection, deep learning, healthcare innovation, diagnostic imaging

## Abstract

**Highlights:**

**What are the main findings?**

- AI-based diagnostic tools demonstrated higher accuracy, sensitivity, and specificity in early breast cancer detection compared to traditional methods.
- Applications in mammography and ultrasound reduced radiologists’ workload and unnecessary procedures, especially in women with dense breast tissue.

**What are the implications of the main findings?**

- Synthesizes recent evidence on AI-assisted breast cancer screening and diagnostic performance.
- Highlights the need for large-scale clinical trials for safe and effective integration into routine screening.

**Abstract:**

**Background:** Breast cancer (BC) remains one of the leading causes of cancer-related deaths worldwide, with early detection being essential for improving survival rates, treatment outcomes, and preventive women’s healthcare strategies. Artificial Intelligence (AI), particularly deep learning (DL) and machine learning (ML) algorithms, has emerged as a promising tool for improving the accuracy and efficiency of BC diagnosis. This systematic review explores the role of AI in early BC detection and its implications for preventive and patient-centered women’s healthcare. **Methods:** A comprehensive search was conducted in PubMed and Scopus for studies published between January 2015 and December 2025, following PRISMA guidelines. The search strategy included combinations of MeSH terms and free-text keywords related to artificial intelligence, machine learning, deep learning, BC screening, mammography, magnetic resonance imaging (MRI), ultrasound, and BC detection. Eleven studies involving approximately 148,170 participants were included. Methodological quality was assessed according to study design. **Results:** AI-driven diagnostic systems demonstrated improved accuracy, sensitivity, specificity, and efficiency compared with conventional approaches. AI applications in mammography and ultrasound reduced radiologists’ workload and healthcare costs while enhancing cancer detection rates, particularly in women with high breast density. AI models also showed potential in identifying metastases and predicting clinical outcomes, supporting more efficient patient management and follow-up care. **Conclusions:** AI-based tools represent a promising advancement in BC detection and screening efficiency. Their integration into BC screening programs may strengthen preventive women’s healthcare services and improve patient outcomes. However, further large-scale clinical validation and real-world implementation studies are required before widespread clinical implementation.

## 1. Introduction

Breast cancer (BC) remains one of the leading causes of cancer-related deaths worldwide, with early detection being a critical factor in improving survival rates and treatment outcomes [1,2]. The International Agency for Research on Cancer (IARC) reports that, in just one year, an estimated 2.3 million new cases of BC were diagnosed worldwide, accounting for 11.7% of all new cancer cases, and that BC was responsible for about 685,000 deaths, or 6.9% of all cancer deaths in females [3].

Over the years, advancements in screening technologies such as mammography, ultrasound, and magnetic resonance imaging (MRI) have significantly improved the ability to identify BC at earlier stages [4,5]. However, challenges remain in reducing false-negative results and ensuring the effectiveness of screening procedures, especially in resource-limited environments [6].

Despite the widespread implementation of mammography-based screening programs, several limitations continue to affect diagnostic performance. False-negative results may delay diagnosis, particularly among women with dense breast tissue, whereas false-positive findings may lead to unnecessary imaging procedures, biopsies, patient anxiety, and increased healthcare costs [4,5,6]. These challenges, together with increasing screening demands, highlight the need for more efficient and reliable diagnostic approaches. Consequently, there is growing interest in innovative technologies that can improve diagnostic accuracy, reduce diagnostic variability, and optimize healthcare resource utilization while maintaining high-quality patient care.

The use of artificial intelligence (AI) in BC diagnosis and detection has garnered significant interest recently. AI, particularly deep learning (DL) and machine learning (ML) algorithms, has emerged as a promising tool for improving the accuracy and efficiency of BC diagnosis. These advanced technologies can analyze complex medical images, learn from vast amounts of data, and provide diagnostic insights that support or surpass traditional methods [7,8].

The integration of AI into BC screening pathways may contribute to more accessible, efficient, and patient-centered preventive women’s healthcare services. Furthermore, AI-assisted screening approaches may support earlier clinical interventions and optimize healthcare resources, particularly in settings with limited radiological capacity. This systematic review examines the role of AI in BC detection, investigating its current capabilities, advantages over conventional diagnostic techniques, and potential issues that need to be addressed for its widespread clinical use.

While previous reviews have primarily focused on the diagnostic performance of AI algorithms, comparatively less attention has been given to the integration of AI applications within preventive and patient-centered women’s healthcare. Therefore, the present review synthesizes current evidence on AI-assisted BC detection across imaging modalities and discusses its potential implications for screening efficiency, clinical decision-making, and healthcare resource utilization. Accordingly, this review evaluates the current evidence on the effectiveness, clinical utility, and potential role of AI-assisted approaches in the early detection of BC in contemporary screening practice.

## 2. Material and Methods

### 2.1. Searching Strategy

A systematic literature search was conducted in electronic databases, including PubMed and Scopus, from January 2015 to December 2025, to identify relevant published articles on recent advances in the early detection of BC using AI. The search strategy was developed using combinations of Medical Subject Headings (MeSH), free-text keywords, and Boolean operators related to AI and BC detection, including: (“artificial intelligence” OR “machine learning” OR “deep learning”) AND (“breast cancer” OR “breast neoplasm” OR “breast carcinoma”) AND (“detection” OR “screening” OR “diagnosis”). Filters were applied to include only peer-reviewed studies published in English between 2015 and 2025. The final database search was conducted on 20 December 2025. This systematic review was conducted in accordance with the Preferred Reporting Items for Systematic Reviews and Meta-Analyses (PRISMA) 2020 guidelines to ensure methodological transparency and reproducibility. No review protocol was prepared or registered prior to conducting this systematic review [9]. Given the heterogeneity of the included studies in study design, AI methodologies, and imaging modalities, a narrative synthesis approach was used to summarize and interpret the findings, without formal subgroup categorization. In addition to the formal systematic review process, selected meta-analyses were examined separately to facilitate broader interpretation of the findings. These publications were chosen for their direct relevance to AI-assisted diagnostic applications, their synthesis of extensive evidence, and their ability to contextualize findings from the included BC studies. They were not included in the PRISMA-guided study selection process and were considered solely for contextual and comparative discussion.

### 2.2. Eligibility Criteria

The studies eligible for inclusion in this systematic review are primarily clinical trials (randomized and case–control studies) and observational studies (retrospective and prospective). The systematic review focuses on studies involving individuals, particularly women, who underwent BC screening or diagnostic tests, regardless of age, ethnicity, or geographic background.

Included studies focused on AI-based tools used for BC detection. This includes ML, DL, and other AI techniques applied to mammography, MRI, ultrasound, and other relevant imaging methods. The outcomes of interest for this systematic review are those that report the sensitivity, specificity, accuracy, early detection rates, or diagnostic performance of AI. Clinical outcomes such as early-stage cancer detection, false-positive and false-negative rates, metastasis detection, tumor characteristics, and survival outcomes were also considered. Additionally, studies comparing the effectiveness of AI against traditional screening methods, such as mammography, are included. Only peer-reviewed studies published in English are considered, and to ensure the relevance of the systematic review, studies published within the last 10 years are included.

### 2.3. Exclusion Criteria

Studies that do not meet the above criteria are excluded from the systematic review. These include editorials, letters to the editor, reviews, and opinion articles that do not present original research data. Studies that focus on populations outside the scope of BC detection, such as non-human subjects or diseases other than BC, are excluded. Additionally, studies that do not focus on AI, such as those centered on traditional mammography without AI, are excluded.

Furthermore, studies published in languages other than English are not considered unless an English translation or abstract is available. Lastly, studies published more than 10 years ago are excluded, as they may not reflect current advancements in AI.

## 3. Results

Initially, 49 studies were identified, 25 from PubMed and 24 from Scopus. After removing eight duplicate records, 41 studies remained for screening. To reduce selection bias and enhance methodological rigor, the screening and eligibility assessment processes were conducted independently by two reviewers using a standardized data extraction form. Extracted information included study characteristics, AI methodology, study population, and diagnostic outcomes. The outcomes of interest included diagnostic performance measures of AI models for BC detection, such as sensitivity, specificity, accuracy, area under the curve (AUC), and cancer detection rates. Additional variables extracted included study design, sample size, imaging modality, type of AI model, and funding sources when reported. When information was missing or unclear, data were extracted as reported in the original studies without imputation, and no assumptions were made. Given the narrative nature of the synthesis, no quantitative effect measures were used. Any disagreements regarding study inclusion were resolved through discussion and consensus. One study was excluded based on the title and abstract evaluation. Of the 40 studies sought for retrieval, one could not be accessed due to the absence of a full text, leaving 39 studies for eligibility assessment.

During eligibility assessment, 28 studies were excluded for various reasons: AI applied in treatment rather than detection (1 study), use of statistical/computational methods instead of direct AI application (8 studies), reviews or opinion pieces (15 studies), inclusion of non-BC patients, but patients with melanoma (1 study), redundancy with an already included clinical trial (2 studies), conducted before 2015 (1 study).

**Figure 1:**
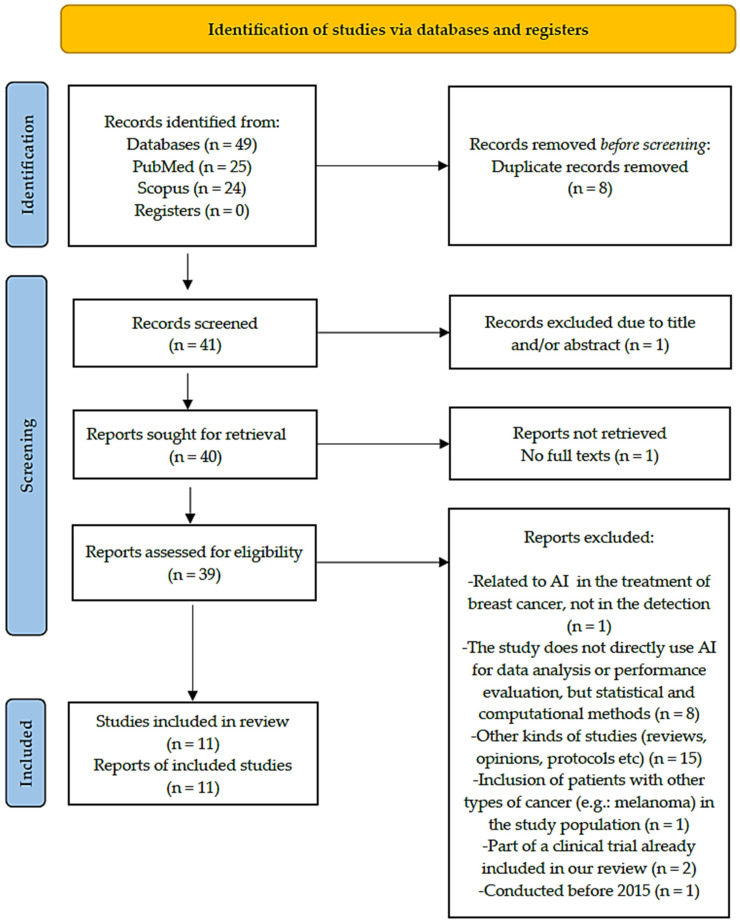
Flow chart of identification, screening, and eligibility of the included studies.

Ultimately, 11 studies were included in the final systematic review (Figure 1 and Table 1). The total number of participants across all studies was approximately 148,170. The included studies varied considerably in sample size, ranging from smaller exploratory investigations to large population-based screening trials, reflecting the diversity of evidence currently available in this field. Given the heterogeneity of the included studies in imaging modalities, clinical objectives, and AI applications, the findings were synthesized narratively and presented in chronological order. The included studies encompassed AI applications in mammography-based screening and detection, MRI and ultrasound-assisted diagnosis, alternative imaging modalities, and predictive models for metastasis, prognosis, and clinical outcomes. The AI methodologies employed included convolutional neural networks, random forest models, and other ML and DL approaches.

More specifically, Salim et al. [10] evaluated an AI-based tool to identify women with a negative mammogram who may still benefit from supplemental MRI screening. The randomized clinical trial (ScreenTrustMRI-NCT04832594) study initially screened 59,354 women, of whom 4103 were eligible to participate based on a high AI score (top 6.9%). Due to an invitation error, 3821 women were invited to join the trial. Among them, 1315 (34%) accepted the invitation, and 663 (50%) were randomized to undergo MRI. Out of those, 559 women (84%) completed the MRI examination, forming the final analysis sample for this study. The results showed that the AI-based triage method detected significantly more cancers than traditional breast density measures, with greater efficiency. The study concluded that using AI to select individuals for supplemental MRI after a negative mammogram improves cancer detection rates and is cost-effective, making it comparable to routine screening mammography.

Van Dooijeweert et al. [11] conducted a non-randomized, single-center clinical trial (International Standard Randomized Controlled Trial Number:14323711) to assess the use of AI assistance for detecting BC metastases in sentinel lymph nodes (SLNs). The study included 190 SN specimens, with 100 in the intervention arm and 90 in the control arm. The study found that AI assistance significantly reduced the need for immunohistochemistry (IHC), resulting in cost savings of ~3000 €, reduced diagnostic time, and a 30% improvement in pathologists’ sensitivity. The trial demonstrated that AI can improve the efficiency and cost-effectiveness of diagnosing metastases in SNs while maintaining safety.

Dembrower et al. [12] assessed the impact of AI on cancer detection and false-positive findings in screening mammography. This prospective clinical trial was conducted at Capio Sankt Göran Hospital in Stockholm and compared AI-assisted reading with standard two-radiologist double reading. The total number of participants in the ScreenTrustCAD study was 55,581 women aged 40–74 who underwent mammography screening. Key findings included that double reading by one radiologist plus AI was non-inferior to the standard double reading, with a slightly higher cancer detection rate (1.04 relative proportion). Additionally, single reading by AI and triple reading with AI were also non-inferior to two-radiologist double reading. The study suggests that AI could be integrated into real-world screening with potential benefits for controlled implementation in practice.

Wang et al. [13] highlighted the potential of using an AI system based on mobile phone-based infrared thermography (IRT) for BC screening, particularly in areas with limited access to traditional screening methods. The study evaluated the performance of the AI-IRT system in 2202 patients, finding it had higher accuracy (0.8627) in detecting BC risk than human readers (0.8088). The study concluded that the AI-IRT system could improve BC screening and increase screening rates, reducing the need for human interpretation.

AI also shows promise in improving the prognosis and follow-up care of BC patients. Specifically, Jin et al. (2023) [14] developed and evaluated two ML models to predict post-treatment events in BC patients, such as tumor recurrence, secondary malignancies, or death. The study used data from 315 patients who experienced stable disease (SD) or progressive disease (PD) after neoadjuvant chemotherapy (NAC) and developed ML models to predict these events. The results showed that the random forest (RF) model outperformed the logistic regression model regarding sensitivity, specificity, and area under the characteristic curve. The RF model performed well in predicting events in BC patients after NAC treatment, helping improve follow-up care and prognosis management.

Fukuda et al. [15] demonstrated the potential of DL using convolutional neural networks (CNNs) in improving the diagnostic accuracy of BC screening. The study included 245 breast masses (146 benign and 99 malignant) from 239 consecutive patients. The results indicated that the AI model outperformed traditional methods, such as the fat-to-lesion ratio (FLR) and elasticity score used in ultrasound elastography, with fewer false positives and a significant increase in positive predictive value. This suggests that AI could improve BC screening, enhance early detection, and reduce unnecessary procedures.

Moreover, AI’s role in improving diagnostic efficiency while reducing healthcare costs is a recurring theme in recent studies. Both Lång et al. in 2023 [16] and Bao et al. in 2023 [17] emphasize the role of AI in enhancing BC detection in mammography, focusing on improving diagnostic performance and reducing radiologists’ workload. More specifically, Bao et al. in 2023 [17] aimed to evaluate radiologists’ diagnostic performance for BC detection, with and without AI support. Six hundred forty-three mammograms were randomly allocated to two groups: one with AI support and the other without. Radiologists in both groups assessed the images, and the results showed that AI support significantly improved diagnostic accuracy. The average area under the receiver operating characteristic curve (AUC) increased from 0.84 to 0.91 with the addition of AI support. Additionally, sensitivity improved from 84.77% to 95.07%, while specificity slightly decreased. The study also found that AI reduced reading time, and overall agreement and performance metrics were better in the AI-supported group. This suggests that AI can enhance radiologists’ diagnostic abilities, improving sensitivity and reducing reading time.

Similarly, a randomized controlled trial by Lång et al. [16] assessed the clinical safety and effectiveness of AI-supported mammography screening compared to standard double reading by radiologists. The study involved 80,033 women, with those in the AI group showing a cancer detection rate of 6.1 per 1000 participants, higher than the control group’s 5.1 per 1000. The AI group also had a higher positive predictive value for recalls (28.3% vs. 24.8%) and reduced the screen-reading workload by 44.3%. The results suggested that AI-supported screening is safe and effective, reducing radiologists’ workload, and that further follow-up is planned to assess long-term outcomes.

Both studies highlight the potential of AI to enhance BC detection. However, Lang et al. emphasize the practical benefits of AI in reducing radiologists’ workload, while Bao et al.’s study focuses on AI’s role in enhancing diagnostic accuracy when used with human expertise.

Moreover, a retrospective case–control study from Salim et al. in 2023 [18] of 714 BC cases and 8029 healthy controls evaluated diagnostic errors by AI, the first radiologist (RAD 1), and the second radiologist (RAD 2) in single- and double-reader settings. The results highlighted that AI significantly reduced false-negative errors when integrated with radiologists, especially in women over 55 and those with high breast density. Additionally, while false-positive assessments were slightly reduced with AI, the most significant improvements in accuracy were seen in older women and those with high breast density. In conclusion, AI has the potential to complement radiologists in BC screening, enhancing diagnostic sensitivity, particularly for high-density and older females.

Liu et al. [19] developed a DL model to predict the malignancy of BI-RADS 4 microcalcifications in breast mammography. The model combined mammography images and clinical data and was tested on 384 patients with 414 pathologically confirmed microcalcifications (221 malignant and 193 benign). The model outperformed traditional clinical models, DL-based image models, and the BI-RADS system, achieving an AUC of 0.910 and demonstrating superior diagnostic accuracy. AI assistance also significantly boosted the performance of junior radiologists, improving their accuracy and interobserver agreement [19].

Bhattarai et al. [20] developed the Surr-INVIGOR model, which used ML to predict the rate of BC growth during the pre-diagnostic stage. The study comprised 114 BC patients aged between 50 and 70 years. The model was based on data from two consecutive mammograms and routine biomarkers. It stratified tumors into fast-growing and slow-growing categories. Patients with fast-growing tumors showed significantly poorer survival. This model facilitates early prognosis assessment and enhances clinical decision-making, providing valuable insights into the potential outcomes for BC patients.

Based on the findings of these studies, AI integration in BC detection and diagnosis holds significant promise. AI-enhanced systems, particularly DL and ML models, have consistently demonstrated improved diagnostic accuracy, sensitivity, and efficiency compared to traditional methods. Several studies have demonstrated that AI-assisted mammography, ultrasound, and MRI systems can outperform human radiologists in sensitivity and specificity, leading to more accurate cancer detection.

Furthermore, AI systems have been shown to reduce radiologists’ workload, enhance cancer detection rates, particularly in women with high breast density, and potentially lower healthcare costs by minimizing the need for costly procedures such as immunohistochemistry. AI’s ability to assist in triaging mammograms, detecting lymph node metastases, and predicting tumor recurrence and post-treatment outcomes contributes to more efficient patient management and improved follow-up care.

Despite these advancements, some studies also highlight the importance of continued research and validation of AI-based tools to ensure their long-term effectiveness and safety in clinical practice. The integration of AI into routine screening may substantially improve BC screening and diagnostic workflows, enhancing both diagnostic performance and operational efficiency. However, further large-scale trials and research are needed to confirm the scalability and sustainability of these technologies in everyday clinical settings.

These findings highlight the potential contribution of AI-assisted screening to preventive and patient-centered women’s healthcare services.

### 3.1. Risk of Bias

**Figure 2:**
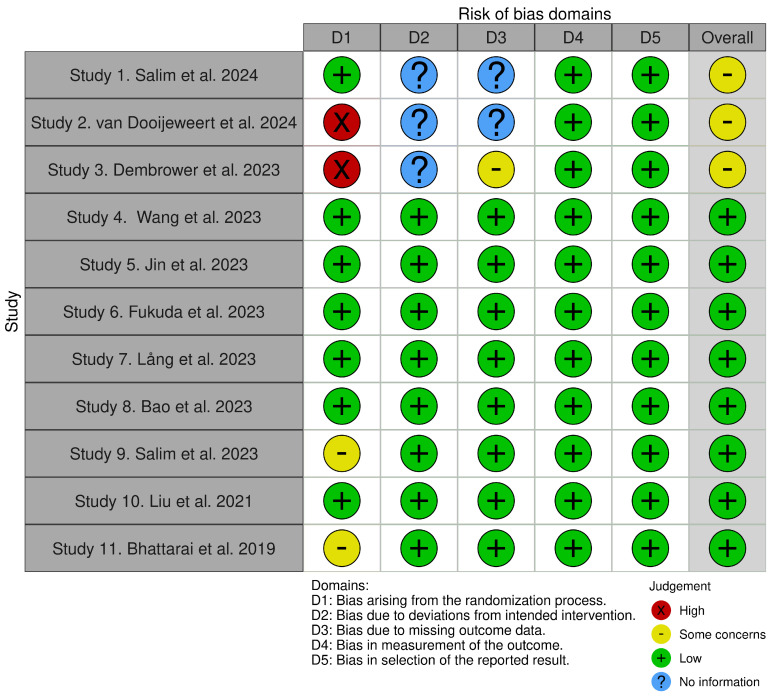
Risk of bias table [10,11,12,13,14,15,16,17,18,19,20].

RoB 2 (Risk of Bias 2) [21] was used as a general framework for assessing the methodological quality of the included studies, particularly randomized controlled trials. The risk-of-bias assessment was independently performed by two reviewers. Any disagreements between the reviewers were resolved through discussion and consensus. Given the heterogeneity of the included study designs, the quality assessment was interpreted narratively according to study characteristics, methodological rigor, and reported outcomes (Figure 2). However, a few studies [11,12,18,20] raised some concerns, mainly due to issues in the randomization process (D1) and missing outcome data (D3). These concerns suggest the need for careful interpretation of their results. Overall, the majority of the included studies demonstrated acceptable methodological quality and consistent findings.

### 3.2. Limitations

This systematic review has some limitations. Only peer-reviewed English-language publications from the last 10 years were included, which may introduce potential language and publication bias. Although PubMed and Scopus capture a substantial proportion of the biomedical literature relevant to BC screening, future reviews may benefit from incorporating additional specialized databases. Finally, no meta-analysis or quantitative synthesis was conducted due to substantial heterogeneity among studies, and the findings were therefore summarized narratively. In addition to these methodological considerations, the included studies were heterogeneous in design, populations, and AI techniques, preventing meta-analysis and limiting direct comparisons. Moreover, many studies were single-center or had relatively small sample sizes, which limits generalizability. Finally, most research was performed in high-resource settings, so the applicability of AI tools in real-world or low-resource environments remains uncertain.

## 4. Discussion

Conventional BC screening techniques, including mammography, ultrasound, MRI, and digital breast tomosynthesis (DBT), remain essential tools for early diagnosis. However, these approaches may be associated with limitations, including false-positive findings, inter-reader variability, and reduced sensitivity in women with dense breast tissue. In recent years, AI, particularly ML and DL algorithms, has emerged as a promising approach to improve diagnostic performance and support radiologists in clinical decision-making. To place the findings of the present systematic review within the broader context of AI-assisted diagnostics, selected meta-analyses were additionally examined. As summarized in Table 2, these publications provide a higher-level overview of current evidence and largely support the observations of the included BC studies, contributing to a more comprehensive understanding of the evolving role of AI in diagnostic medicine.

Li et al. [22] conducted a meta-analysis on the diagnostic performance of DL algorithms combined with ultrasound for BC diagnosis. The study included data from 20 studies published between 2017 and 2023, comprising 14,955 cases, including both BC patients and those with benign lesions. The study found that DL and multimodal ultrasound fusion significantly improved diagnostic accuracy. The multimodal ultrasound group performed better than the B-mode ultrasound group, with a higher sensitivity (0.93 vs. 0.92) and AUC (0.787 vs. 0.642). These findings highlight the superior diagnostic performance of DL combined with ultrasound for BC detection.

Xavier et al. [23] investigated whether AI-based triaging of BC screening mammograms could reduce radiologists’ workload without compromising sensitivity. A meta-analysis of 13 studies revealed that AI reduced workload by 68.3%, with a sensitivity of 93.1% and a specificity of 68.7%. This suggests that AI can optimize healthcare resources while maintaining high diagnostic accuracy.

Tabnak et al. [24] reviewed the diagnostic accuracy of MRI-based radiomics, including DL algorithms, for predicting Ki-67 expression in BC. The results demonstrated good diagnostic performance, with a pooled sensitivity of 0.80, specificity of 0.82, and an AUC of 0.88 in training cohorts. However, the sensitivity and specificity did not exceed 90%, limiting its use as a supplement to current diagnostic methods, such as biopsy or surgery.

Liu et al. [25] systematically reviewed the diagnostic accuracy of ML methods for mammography-based BC diagnosis. A total of 32 studies, comprising 23,804 images, were included. The results showed high performance, with an overall sensitivity of 91.4%, specificity of 91.6%, and AUC of 0.945. CNNs performed the best, with a sensitivity of 96.1%, specificity of 95.0%, and an AUC of 0.974, supporting the potential of ML and CNNs to enhance BC screening through mammography.

Yoon et al. [26] evaluated the performance of AI systems in interpreting digital mammography and DBT. They analyzed 16 studies with 1,108,328 examinations. AI outperformed radiologists in reader studies on digital mammography, with a higher AUC (0.87 vs. 0.81), and also performed better in DBT interpretation (AUC 0.90 vs. 0.79). However, AI showed higher sensitivity but lower specificity. This suggests that AI can perform as well as or better than radiologists in digital mammography; however, further research is needed to define AI’s role in DBT.

Liang et al. [27] reviewed the diagnostic accuracy of ML models combined with MRI in predicting the pathological response to neoadjuvant chemotherapy in BC patients. Seventeen studies with 3392 patients were included in their research. DL algorithms outperformed ML methods, with an AUC of 0.92 versus 0.87. The study concluded that DL offers higher performance, especially when combined with MRI and clinical or histopathologic data.

Hickman et al. [28] evaluated stand-alone ML algorithms within mammographic screening workflows, reporting a pooled sensitivity of 75.4%, specificity of 90.6%, and AUC of 0.89. These results indicate that ML algorithms can match or exceed human reader performance, enhancing efficiency in mammography screening.

Compared with conventional radiological assessment, AI-based systems generally demonstrate comparable or improved diagnostic performance across BC screening modalities. Several included studies have reported higher AUC, sensitivity, and overall accuracy for AI-assisted approaches compared to traditional radiologist interpretation, particularly in mammography and DBT. In addition, AI systems have shown potential to reduce radiologists’ workload while maintaining diagnostic performance, suggesting a complementary rather than replacement role in clinical practice. Several studies further demonstrated that AI applications show promising results across both diagnostic and prognostic domains. Diagnostic AI systems were primarily used for cancer detection, image interpretation, and differentiation between benign and malignant lesions, whereas prognostic AI models focused on metastasis identification, recurrence prediction, and clinical outcome assessment.

Across the included studies, AI-assisted approaches consistently achieved high diagnostic performance. Reported sensitivity values ranged from approximately 84.8% to 95.1%, while specificity values generally exceeded 90% in several studies, and AUC values frequently surpassed 0.90. For example, Bao et al. reported an increase in AUC from 0.84 to 0.91 and an improvement in sensitivity from 84.8% to 95.1% with AI support [17], whereas Liu et al. reported an AUC of 0.910 for predicting malignant microcalcifications using a DL model [19]. Similarly, AI-supported mammography screening demonstrated higher cancer detection rates and reduced radiologist workload in the study by Lång et al. [16].

These findings have important implications for clinical practice, particularly given the increasing demand for BC screening services and the limited availability of experienced breast radiologists in some healthcare settings. By automatically prioritizing examinations, assisting lesion detection, and reducing the number of cases requiring double reading, AI systems may help optimize radiologists’ workload while maintaining diagnostic performance. For example, Xavier et al. reported a 68.3% reduction in workload, whereas Lång et al. demonstrated a 44.3% reduction in screen-reading workload without compromising cancer detection rates [16,23]. Such improvements may increase screening capacity and support more efficient utilization of healthcare resources.

While most included studies focused on diagnostic accuracy, several findings may also be relevant to broader screening outcomes. Improved detection following negative mammography [10], reduced false-negative findings in women with dense breast tissue [18], fewer unnecessary procedures [15], and improved recall performance [16] suggest that AI may influence clinically relevant aspects of the screening pathway beyond sensitivity and specificity alone. However, outcomes such as interval cancer rates, overdiagnosis, and recall-related anxiety were only rarely reported in the available literature.

Breast density remains a significant challenge in BC screening because dense fibroglandular tissue may obscure malignant lesions and reduce mammographic sensitivity. AI-assisted image analysis may help identify subtle imaging patterns and lesion characteristics that can be obscured on mammographic examinations, potentially reducing false-negative findings and improving diagnostic performance [17]. Clinically, this may support earlier diagnosis, reduce the likelihood of missed cancers, and improve screening effectiveness among women with dense breasts. These observations are consistent with recent literature highlighting the growing role of AI in BC screening, risk prediction, and personalized prevention strategies, particularly when integrated with imaging and clinical data [29]. Although one included study evaluated AI performance in BI-RADS 4 microcalcifications [19], breast-density-specific outcomes according to BI-RADS density categories were not consistently reported across the included studies. Consequently, whether AI-derived benefits vary across specific breast density categories remains unclear.

The included studies demonstrated the use of both ML and DL methodologies in BC detection and management. DL approaches, such as convolutional neural networks, have been primarily applied to imaging-based tasks, including mammography, ultrasound, and MRI interpretation, where they have demonstrated high diagnostic performance and improved lesion detection [15,18,19]. In contrast, ML models, including random forest and other predictive algorithms, were more commonly used for prognosis assessment, recurrence prediction, and clinical outcome modeling [14,20]. While DL approaches appear particularly advantageous for complex image analysis, ML models may offer greater interpretability and applicability when structured clinical variables are used. These findings suggest that ML and DL should be viewed as complementary rather than competing approaches within BC care. The interpretation of AI performance should also consider differences in training datasets, annotation practices, and validation strategies across studies. Variability in patient populations, imaging data, and external validation may influence model generalizability and limit direct comparisons between studies.

The studies included in this systematic review applied a wide range of AI methodologies across different BC screening and diagnostic modalities, including mammography, ultrasound, MRI, IRT, elastography, and DBT. ML and DL approaches, particularly CNNs and RF models, were frequently utilized to improve diagnostic accuracy, sensitivity, and clinical decision-making. However, substantial heterogeneity was observed among studies in imaging techniques, AI methodologies, sample sizes, study designs, and clinical settings, which may affect the comparability and generalizability of the findings. Therefore, the reported diagnostic performance metrics should be interpreted with caution, as differences in study populations, data quality, and validation strategies may influence the observed results. The use of AI in BC detection also raises important ethical considerations. Key concerns include patient data privacy and security, particularly due to the use of large imaging datasets. Algorithmic bias may also lead to unequal performance across populations, while the limited interpretability and transparency of some DL models may reduce clinician trust, explainability, and acceptance in routine clinical practice. Furthermore, medico-legal responsibility in cases of diagnostic errors remains unclear. Therefore, ethical guidelines and robust validation are essential for safe clinical implementation. Despite the limitations mentioned above, the current evidence supports continued investigation of AI-based approaches in BC screening and diagnosis, and further large-scale, multicenter studies are needed to confirm their clinical utility and generalizability. Emerging computational paradigms, including quantum ML, have recently been explored for BC prediction and decision support research [30]. However, these approaches remain in their early stages of development, and their potential clinical value has yet to be demonstrated in real-world screening and diagnostic settings.

## 5. Conclusions

In summary, the integration of AI into BC detection and diagnosis shows promise for improving the accuracy and efficiency of screening and diagnostic procedures. AI-assisted systems, including DL and ML models, have shown improved diagnostic performance across imaging modalities such as mammography, ultrasound, and MRI, particularly in women with dense breast tissue. In addition, AI-based approaches may support clinical decision-making by reducing radiologists’ workload and facilitating more efficient triage, metastasis detection, and patient management.

Although current findings are encouraging, further validation through large-scale and multicenter clinical studies remains necessary to ensure the long-term safety, effectiveness, and generalizability of AI-assisted technologies. The integration of AI into BC screening programs may improve operational efficiency, optimize healthcare resources, and support preventive and patient-centered women’s healthcare services. However, additional research is required before widespread routine clinical implementation can be achieved.

## Figures and Tables

**Table 1:** Clinical trials and randomized controlled trials are included in the systematic review.

Study	First Author, Year, Ref. No.	Country	Type of Study	Field of Application of AI	Sample	Results	Conclusion
1	Salim et al. 2024 [10]	Sweden	Randomized clinical trial [ScreenTrust magnetic resonance imaging (MRI)trial (NCT04832594)]	Breast cancer (BC) detection using MRI	559 women	The present artificial intelligence (AI) technique was nearly four times more effective at detecting tumors per 1000 MRI exams (64 versus 16.5) than traditional breast density assessments. This study showed that selecting a small percentage (6.9%) of people for further MRI following negative mammography based on an AI-based score finds many missed tumors, making the cost per cancer discovered comparable to screening mammography.	Promising diagnostic performance
2	van Dooijeweert et al. 2024 [11]	Netherlands	Non-randomized, single-center clinical trial (International Standard Randomized Controlled Trial Number:14323711)	Detection of BC metastases in sentinel lymph nodes (SLNs)	190 (SLN) specimens, with 100 in the intervention arm and 90 in the control arm	AI-assisted pathologists had a significantly lower adjusted relative risk of using immunohistochemistry, resulting in ~3000 € in cost savings. Significant time savings and a 30% increase in sensitivity were demonstrated by secondary endpoints for pathologists using AI. This trial demonstrates that AI support is safe and can save time and money.	Promising diagnostic performance
3	Dembrower et al. 2023 [12]	Sweden	Prospective clinical trial (ScreenTrustCAD, NCT04778670)	BC detection using mammography	55,581 women aged 40–74 years, who underwent mammography screening	When one radiologist was replaced by AI to read screening mammograms independently, the non-inferiority cancer detection rate was 4% higher than when a radiologist performed double reading.	Promising diagnostic performance
4	Wang et al. 2023 [13]	China	Prospective clinical trial (NCT04761211)	BC detection using infrared thermography (IRT) via a mobile phone	2202 patients	The study concludes that the AI-IRT system can enhance BC screening, particularly in areas with limited access to traditional methods, thereby reducing reliance on human interpretation. This system shows promise in enhancing BC diagnosis, reducing reliance on human judgment, and increasing screening availability.	Promising diagnostic performance
5	Jin et al. 2023 [14]	China	Randomized controlled trial	Evaluation of prognosis after neoadjuvant chemotherapy (NAC) for BC	315 patients	The random forest (RF) model performs well at predicting events in BC patients after NAC and may help detect tumor recurrence and improve patient follow-up. Based on the results, machine learning (ML) models, especially RF, could help better manage BC patients by providing useful information for post-treatment prognosis.	Promising diagnostic performance
6	Fukuda et al. 2023 [15]	Japan	Randomized controlled trial	BC detection using ultrasound and elastography	245 breast masses (146 benign and 99 malignant) from 239 consecutive patients	An AI-based convolutional neural network (CNN) model demonstrates superior diagnostic performance in distinguishing benign from malignant breast masses compared with traditional methods, such as the fat-to-lesion ratio (FLR) and elasticity scoring. The model is more accurate, with fewer false positives, and significantly increases positive predictive value. This suggests that AI could play a crucial role in improving the accuracy and efficiency of BC screenings by reducing unnecessary procedures and enhancing early detection.	Promising diagnostic performance
7	Lång et al. 2023 [16]	Sweden	Randomized controlled trial (NCT04838756)	BC detection using mammography	80,033 women	The use of AI in mammography screening is both safe and effective. The AI-supported screening yielded a cancer detection rate comparable to that of traditional double reading by radiologists, with the added benefit of significantly reducing radiologists’ workload. This is important because it suggests that AI can enhance efficiency and maintain accuracy in detecting cancers, potentially leading to a more streamlined screening process.	Promising diagnostic performance
8	Bao et al. 2023 [17]	China	Observational retrospective study	BC detection using mammography	643 mammograms	The findings suggest that AI can enhance radiologists’ diagnostic abilities, particularly by improving sensitivity and reducing reading time; however, further improvements in AI algorithms and prospective studies are needed.	Promising diagnostic performance
9	Salim et al. 2023 [18]	Sweden	Retrospective case–control study	BC detection using mammography	714 BC cases and 8029 healthy controls	AI has the potential to complement radiologists in BC screening, enhancing diagnostic sensitivity, particularly for high-density and older females.	Promising diagnostic performance
10	Liu et al. 2021 [19]	China	Randomized controlled trial	BC detection using mammography, especially in microcalcifications	384 patients with 414 pathologically confirmed microcalcifications (221 malignant and 193 benign)	The deep learning (DL) model demonstrated high diagnostic power, sensitivity, and specificity for predicting malignant BI-RADS 4 microcalcifications. It demonstrated similar performance to that of senior radiologists and outperformed junior radiologists. AI-assisted diagnosis improved diagnostic performance and interobserver agreement, supporting more accurate clinical decisions.	Promising diagnostic performance
11	Bhattarai et al. 2019 [20]	UK & USA	Clinical trial	Prediction of BC growth rate in vivo	114 BC patients aged between 50 and 70 years	The model categorizes tumors as either fast-growing or slow-growing, providing valuable insights into overall survival for patients. Patients with fast-growing tumors showed significantly poorer survival. Surr-INVIGOR can aid in early prognosis assessment and improve clinical decision-making.	Promising diagnostic performance

**Table 2:** Meta-analyses investigated AI in BC detection.

Ref. No.	First Author, Year	Field of Application of AI	Results	Conclusion
22	Li et al. 2024 [22]	BC detection using ultrasound	Combining DL with ultrasound significantly improves BC detection, and fusing DL with multimodal ultrasound yields superior diagnostic performance compared to B-mode ultrasound alone.	Promising diagnostic performance
23	Xavier et al. 2024 [23]	BC detection using mammography	This study evaluated whether AI-based triaging of BC screening mammograms could reduce radiologists’ workload without compromising sensitivity. A meta-analysis of 13 studies revealed that AI reduced workload by 68.3%, with a sensitivity of 93.1% and a specificity of 68.7%.	Promising diagnostic performance
24	Tabnak et al. 2024 [24]	MRI-based radiomics (including deep learning) for predicting Ki-67 expression in BC	This study assessed the diagnostic accuracy of MRI-based radiomics, including DL, for predicting Ki-67 expression in BC. While MRI-based radiomics has demonstrated promise, its sensitivity and specificity do not exceed 90%, limiting its use as a supplement to current diagnostic methods, such as biopsy or surgery.	Promising diagnostic performance
25	Liu et al. 2023 [25]	BC detection using mammography	Meta-analysis of the diagnostic accuracy of ML methods for mammography-based BC diagnosis. A total of 32 studies, comprising 23,804 images, were included. The results showed high performance, with an overall sensitivity of 0.914, specificity of 0.916, and area under the receiver operating characteristic curve (AUC) of 0.945. Among the ML methods, CNN performed the best, with a sensitivity of 0.961, specificity of 0.950, and AUC of 0.974. The study concludes that AI-based ML methods, especially CNNs, show excellent potential to improve BC screening through mammography.	Promising diagnostic performance
26	Yoon et al. 2023 [26]	BC detection using mammography and digital breast tomosynthesis (DBT)	This study analyzed 16 studies with 1,108,328 examinations. The study concluded that AI performs as well as or better than radiologists in digital mammography; however, more research is needed to determine AI’s role in DBT.	Promising diagnostic performance
27	Liang et al. 2022 [27]	Diagnostic accuracy of ML models combined with MRI in predicting the pathological response to NAC in BC	Seventeen studies with 3392 patients were included. The results showed that ML and MRI had a moderate accuracy (AUC = 0.87), while DL algorithms performed better (AUC = 0.92). Additionally, studies that combined MRI with clinical or histopathologic data outperformed those using MRI alone. The study concluded that ML with MRI achieves moderate predictive accuracy, whereas DL achieves higher performance.	Promising diagnostic performance
28	Hickman et al. 2022 [28]	BC detection using mammography	These results demonstrate that ML can match or exceed human reader performance, improving efficiency in mammography screening.	Promising diagnostic performance

## Data Availability

No new data were created or analyzed in this study.

## Abbreviations

The following abbreviations are used in this manuscript:

AUC	Area Under the Curve
AI	Artificial Intelligence
BC	Breast Cancer
CNNs	Convolutional Neural Networks
DBT	Digital Breast Tomosynthesis
DL	Deep Learning
FLR	Fat-to-Lesion Ratio
IARC	International Agency for Research on Cancer
IHC	Immunohistochemistry
IRT	Infrared Thermography
ML	Machine Learning
MRI	Magnetic Resonance Imaging
NAC	Neoadjuvant Chemotherapy
PD	Progressive Disease
PRISMA	Preferred Reporting Items for Systematic Reviews and Meta-Analyses
RF	Random Forest
RCTs	Randomized Controlled Trials
SD	Stable Disease
SLNs	Sentinel Lymph Nodes
